# First report of a gastropod parasitic nematode *Phasmarhabditis californica* (Nematoda: Rhabditidae) in Alberta, Canada

**DOI:** 10.21307/jofnem-2020-092

**Published:** 2020-08-25

**Authors:** Taylor Brophy, Dana K. Howe, Dee R. Denver, Lien T. Luong

**Affiliations:** 1 Department of Biological Sciences, University of Alberta, Edmonton, AB, Canada; 2 Department of Integrated Biology, Oregon State University, Corvallis, OR

**Keywords:** Alberta, *Arion rufus*, Biological control, Canada, Slug, PCR, *Phasmarhabditis*

Invasive European slugs damage a range of crops in North America, including canola, soybean, maize, wheat, strawberries, asparagus, Brussels sprouts, and ornamental plants (Barker, 2002). The status of slug and snail as pests has been growing globally, yet the widespread use of chemical control measures is less than ideal (Bailey, 2002; Wilson and Rae, 2015). Baited pellets and liquid sprays containing methiocarb, metaldehyde, iron phosphate, and sodium ferric EDTA sometimes fail to provide adequate protection for crops (Hata et al., 1997; Bailey, 2002). These products are also poisonous to birds, mammals, and invertebrates (Purves and Bannon, 1992; Fletcher et al., 1994; Bailey, 2002). In Europe and the UK, a biological control agent, *Phasmarhabditis hermaphrodita* (Nematoda: Rhabditidae) against slugs and snails has been formulated into a biological molluscicide (Nemaslug^®^) for horticultural and agricultural application (Glen and Wilson, 1997). This nematode is a facultative parasite that can complete its life cycle without a host; however infective stages can penetrate and infect slugs and snails. The larvae feed on the host and develop into adults, which are hermaphrodites and primarily oviparous (Maupas, 1900). The nematodes kill the host within 4 to 21 days and eventually produce a new generation (Glen and Wilson, 1997; Tan and Grewal, 2001). To date, no *Phasmarhabditis* species has been found in Canada, though a few species have been discovered in the United States, including *P. hermaphrodita,* plus two new species, *P. californica* and *P. papillosa* (Tandingan De Ley et al., 2016; Mc Donnell et al., 2018). These new finds have spurred interest in identifying a natural biological control agent against slugs in Canada.

## Methods and results

A total of 2,406 slugs were collected from residential gardens and local nurseries during a survey in the greater area of Edmonton. Nine slug species (*Arion fasciatus, A. hortensis, A. rufus, Prophysaon andersoni, Deroceras reticulatum, D. invadens, D. leave, Ambigolimax valentianus,* and *Limax maximus*) were identified, representing three slug families (Arionidae, Agriolimacidae, and Limacidae). Slugs were returned to the lab and exterminated by severing the head, and placed individually in a petri dish lined with a moistened filter paper. The dishes were then incubated at 12 hr light, 18°C, 80% RH, and 12 hr dark, 12°C, 60% RH. The slugs were monitored over the course of two weeks for the presence of nematodes. Nematode samples were taken in triplicate and stored in 95% EtOH before molecular analysis. We found a putative *Phasmarhabditis* species in association with an *Arion rufus* slug (id verified by R. Mc Donnell, OSU), collected from the exterior grounds of a local nursery. PCR amplification and subsequent direct DNA sequencing of an ~800 bp segment of the nematode 18S ribosomal RNA gene revealed a sequence that was 100% match to the 18S rRNA sequence for *P. californica* (KM510210; Tandingan De Ley et al., 2014, 2016) in GenBank. Our newly generated *P. californica* 18S sequence was submitted to GenBank under accession number MT135094.

## Discussion

This is the first study to report on the occurrence of *P. californica* in Canada. Our results highlight the need for further field surveys in Canada to determine the distribution and abundance of this nematode. The nematode *P. californica* was first described in association with a slug in California (Tandingan De Ley et al., 2016) and has recently been isolated in New Zealand (Wilson et al., 2016) and Europe (Carnaghi et al., 2017). The discovery of *P. californica* in Canada has potentially significant regulatory implications for pest management generally and specifically for biological control against a growing slug problem. Biological control has many advantages over chemical pesticides as it offers greater specificity while using fewer toxic chemicals. For example, *P. hermaphrodita* only attacks slugs and snails, and is harmless to other organisms, such as earthworms, birds, and beneficial insects (Rae et al., 2007). Research on the infectivity and host specificity of this Canadian strain against pestiferous and native gastropods is needed to determine its potential as a biological control agent in Canada. Furthermore, *Phasmarhabditis sp.*, a facultative parasite, could also be employed as new genetic model nematodes to study the evolution of parasitism (Andrus and Rae, 2019; Luong and Mathot, 2019).
